# On the Theory and Practice of Midwifery

**Published:** 1851-08

**Authors:** 


Art. VIII.—*On the Theory and Practice of Midwifery.* By Fleetwood Churchill, M.D., M.R.I.A., etc. With notes and additions, by D. Francis Condie, M.D., Secretary of the College of Physicians, etc., etc. With one hundred and thirty-nine illustrations. A new American, from the last improved Dublin edition. Philadelphia: Blanchard & Lea. 1851.

In noticing a former edition of this work, we placed it among the very best works in the hands of the profession, on the theory and practice of midwifery. Additional observation has confirmed and strengthened our previous convictions. But if the former editions were valuable, the present edition has enlarged its claims to professional favor, for in addition to new contributions from the experience of Dr. Churchill, the American editor, Dr. Condie, has made about seventy notes, the most of which are of great utility and value.

We do not deem it necessary to dwell upon the merits of Dr. Churchill, as a writer on obstetrics, for his fame has extended far beyond any circle of influence we can expect to reach. The *London Medical Gazette* of a recent date, compared this work with that of Professor Miller, of the Louisville University, and placed them in the first rank as obstetrical works. It is gratifying to see the reciprocal feelings of kindness and courtesy that are springing up between the medical minds of England and the United States. The science of medicine is a glorious unit, and its assiduous cultivators should endeavor, upon all occasions, to promote good will and good fellowship among its members. Paltry bickerings and intemperate conduct among those who are looked upon as exponents of scientific truths, are calculated to aid the quack and the charlatan, thus doing injury to the profession and to society. The examples furnished in the treatment of Dr. Churchill's works in this country, and of Professor Miller's book on Human Parturition, in England, are cheering signs of a brighter dawn, and we earnestly hope that this same light will spread until every dark spot in the profession shall be enlightened with its refulgent beams.

This volume is admirably executed in its mechanical department, and we advise such of our readers as wish to purchase obstetrical works of value, to secure Condie's edition of Churchill for their library. B.

